# Association Between Primary vs. Secondary Endodontic Infection and Maxillary Sinus Schneiderian Membrane Thickness on CBCT: A Cross‐Sectional Study

**DOI:** 10.1155/ijod/8232083

**Published:** 2026-09-16

**Authors:** Seyyed Amir Seyyedi, Aisan Ghaznavi, Salar Payahoo, Maryam Rezaeipour, Mohammad Reza Ghorbani Afkhami

**Affiliations:** ^1^ Department of Oral and Maxillofacial Diseases, School of Dentistry, Urmia University of Medical Sciences, Urmia, Iran, umsu.ac.ir; ^2^ Department of Oral and Maxillofacial Radiology, School of Dentistry, Urmia University of Medical Sciences, Urmia, Iran, umsu.ac.ir; ^3^ Student Research Committee, Urmia University of Medical Sciences, Urmia, Iran, umsu.ac.ir; ^4^ Research Assistant, Urmia University of Medical Sciences, Urmia, Iran, umsu.ac.ir

**Keywords:** cone-beam computed tomography, maxillary sinus, nasal mucosa, periapical diseases

## Abstract

**Background and Objective:**

Changes in the maxillary sinus mucosa and increased thickness of the Schneiderian membrane in the vicinity of endodontic lesions are common findings on CBCT. The aim of this study was to compare Schneiderian membrane thickness (SMT) in the maxillary sinus adjacent to primary and secondary endodontic infections and to investigate its relationship with selected radiographic variables.

**Materials and Methods:**

This retrospective cross–sectional study was performed on archival CBCT images. A total of 172 CBCT scans, including 86 cases with primary endodontic lesions and 86 cases with secondary endodontic lesions, were evaluated between September 2023 and March 2024. The study variables included SMT, bone thickness (BT) between the lesion and the sinus floor, lesion volume (LV), mucosal morphology, and alveolar bone perforation. Inter‐rater reliability for quantitative measurements was assessed.

**Findings:**

In the total sample, the mean SMT was 3.72 ± 2.24 mm. Alveolar bone perforation was present in 62 cases. The mean SMT was 3.74 ± 2.01 mm in the primary group and 3.69 ± 2.46 mm in the secondary group. The mean difference was 0.06 mm (95% confidence interval [95% CI]: −0.63 to 0.71 mm), with no significant difference between the groups (*p* = 0.401). No significant correlations were observed between SMT and age, LV, or BT.

**Conclusion:**

In this CBCT study, SMT was not significantly associated with primary versus secondary endodontic lesions. In addition, no significant associations were observed between SMT and age, LV, or BT between the lesion and the sinus floor. These findings should be interpreted as radiographic associations rather than causal relationships.

## 1. Introduction

The paranasal sinuses are air‐filled cavities located within the bones of the skull [1]. Among the paranasal sinuses, the maxillary sinus is the largest and is located in the middle third of the face [2]. The anatomical features associated with the floor of the sinus and its relationship with adjacent structures (especially in the posterior maxilla) are important from a dental perspective [3, 4]. Ventilation and drainage of the maxillary sinus are carried out through the ostium into the nasal cavity, and proposed functions include reduction of skull weight and conditioning of inspired air [5].

The Schneiderian membrane lines the maxillary sinus, and its changes on radiographic imaging, especially in the form of Schneiderian membrane thickening, are frequently reported findings [6]. The normal thickness of the Schneiderian membrane as well as conditions associated with Schneiderian membrane thickening have been investigated in various studies [7, 8]. In some sources, Schneiderian membrane thickness (SMT) exceeding a threshold (e.g., 2 mm) has been described as a radiographic indicator of maxillary sinusitis [9].

Maxillary sinusitis is one of the most common infectious diseases of the sinus area, and a considerable proportion of its cases can have an odontogenic origin [10, 11]. The odontogenic causes of maxillary sinusitis and the importance of its correct diagnosis have been emphasized in various sources [12]. Some reports suggest that odontogenic sinusitis may be delayed in practice and may be diagnosed late in clinical practice, potentially resulting in greater disease burden at the time of diagnosis [13].

Several factors, including microbial infections, fungal agents, and dental conditions or dental treatments, can contribute to the development or exacerbation of maxillary sinusitis [14–16]. Among odontogenic causes, periapical lesions and infections of the apical region of the maxillary posterior teeth can be associated with changes in the sinus mucosa due to their proximity to the sinus floor [7].

Some studies have reported that the presence of periapical lesions is directly related to increased thickness of the Schneiderian membrane [17, 18]. The role of infection spread and adjacent anatomical/pathological conditions (including bone defects) in this relationship has also been considered [19]. Evaluating these changes using more precise imaging techniques may improve the interpretation of findings and support clinical decision‐making [20].

Endodontic infections are usually classified into two categories: “primary” and “secondary” [21]. Primary endodontic infection usually involves microbial colonization of a previously untreated root canal system and can be associated with apical periodontitis [22]. In contrast, secondary endodontic infection generally refers to persistent or recurrent infection in previously treated teeth and is often associated with microorganisms that persist or reenter the canal system after treatment [21]. Biological, particularly microbiological, differences between primary and secondary endodontic infections have been reported in the endodontic literature [23].

From an imaging perspective, two‐dimensional methods have limitations in accurately assessing periapical–sinus relationships, such as overlapping structures [24, 25]. CBCT, as a three‐dimensional method, allows for more accurate assessment of dental and periapical structures and maxillary sinus [20, 26, 27]. The use of CBCT in the assessment of sinus mucosa thickness, periapical lesions, and anatomical connections has been reported in various studies [28]. Compared with conventional two‐dimensional imaging, CBCT may have advantages in terms of adequate resolution for dental structures and the possibility of reconstruction in different planes [29, 30].

Although endodontic lesions have been associated with changes in the sinus mucosa, it is unclear whether the type of endodontic infection (primary vs. secondary) is associated with different patterns of SMT [6, 17, 18]. Clarifying whether SMT differs according to lesion type may help prevent overinterpretation of Schneiderian membrane changes on CBCT and improve radiographic decision‐making in endodontically involved posterior maxillary teeth. Therefore, the aim of the present study was to investigate and compare the SMT of the maxillary sinus adjacent to primary and secondary endodontic lesions based on CBCT images and to assess its association with relevant radiographic variables.

## 2. Methods

### 2.1. Study Design

This study is a retrospective cross–sectional study based on archival CBCT scans. CBCT images were extracted from the Ghaznavi radiology center in Urmia and evaluated from September 2023 to March 2024. All CBCT scans were archival images that had been obtained previously for routine diagnostic purposes as part of patient care and were not acquired specifically for this study or solely for the measurement of SMT.

### 2.2. Sample Size and Sampling Method

Convenience sampling was used. Sample size estimation was performed with reference to the CBCT study by Garcia‐Font et al. [31], which compared SMT between primary and secondary endodontic infections. With a significance level of 0.05 and a statistical power of 80%, the required sample size was calculated as 86 cases per group. Archival CBCT scans were screened sequentially until the prespecified sample size of 172 eligible cases (86 primary and 86 secondary cases) with complete evaluable data was reached. During screening, scans were excluded when image artifacts or technical limitations prevented reliable measurement of the required radiographic variables or when classification as a primary or secondary endodontic lesion could not be established with sufficient confidence. Because case selection continued until the calculated sample size was reached, a detailed screening log with the exact number of excluded scans and reasons for exclusion was not retained.

### 2.3. Ethical Approval

The research protocol was conducted after obtaining approval from the ethics committee (IR.UMSU.REC.1402.236). Given the retrospective use of archival anonymized CBCT scans, no additional radiation exposure was imposed for research purposes. Patient identifiers were removed before analysis, and confidentiality was maintained throughout the study.

### 2.4. Inclusion and Exclusion Criteria

Inclusion criteria comprised CBCT images showing a periapical lesion associated with maxillary posterior teeth (second premolar to second molar) adjacent to the maxillary sinus, with adequate image quality for measurements.

Exclusion criteria were (1) teeth without endodontic/periapical pathology in the study area, (2) third molars, and (3) imaging evidence of acute nonodontogenic sinusitis (e.g., an air–fluid level and/or diffuse Schneiderian membrane thickening involving all sinus walls). To reduce the effect of nonodontogenic factors, the contralateral sinus was also examined to confirm the absence of acute nonodontogenic features (4). CBCT scans with artifacts, technical errors, or other image‐quality limitations that prevented reliable measurement of one or more required radiographic variables; and (5) cases in which classification as a primary or secondary endodontic lesion could not be established with sufficient confidence based on the available imaging findings and treatment history.

### 2.5. Operational Definition of Groups

Cases were classified as primary or secondary endodontic infection/lesion based on CBCT findings and root canal treatment status.

Primary endodontic infection/lesion: Presence of periapical radiolucency in the apical third of the root such that the width of the radiolucency was at least twice the width of the periodontal ligament space in a tooth with no history of root canal treatment.

Secondary endodontic infection/lesion: Presence of periapical radiolucency meeting the same criterion in the apical third, in a tooth with a history of root canal treatment, consistent with persistent/recurrent posttreatment infection.

### 2.6. CBCT Imaging Protocol

All scans were acquired using a Planmeca CBCT unit (Helsinki, Finland). Imaging parameters included 84 kV, 8 mA, and an exposure time of 12 s. The voxel size was set to 0.2 mm. Images were reconstructed in axial, coronal, and sagittal planes with a reconstruction slice thickness of 0.2 mm. The effective dose for the small field‐of‐view protocol was reported to range from 19 to 44 microsieverts.

### 2.7. Measurement Methods

**Figure 1:**
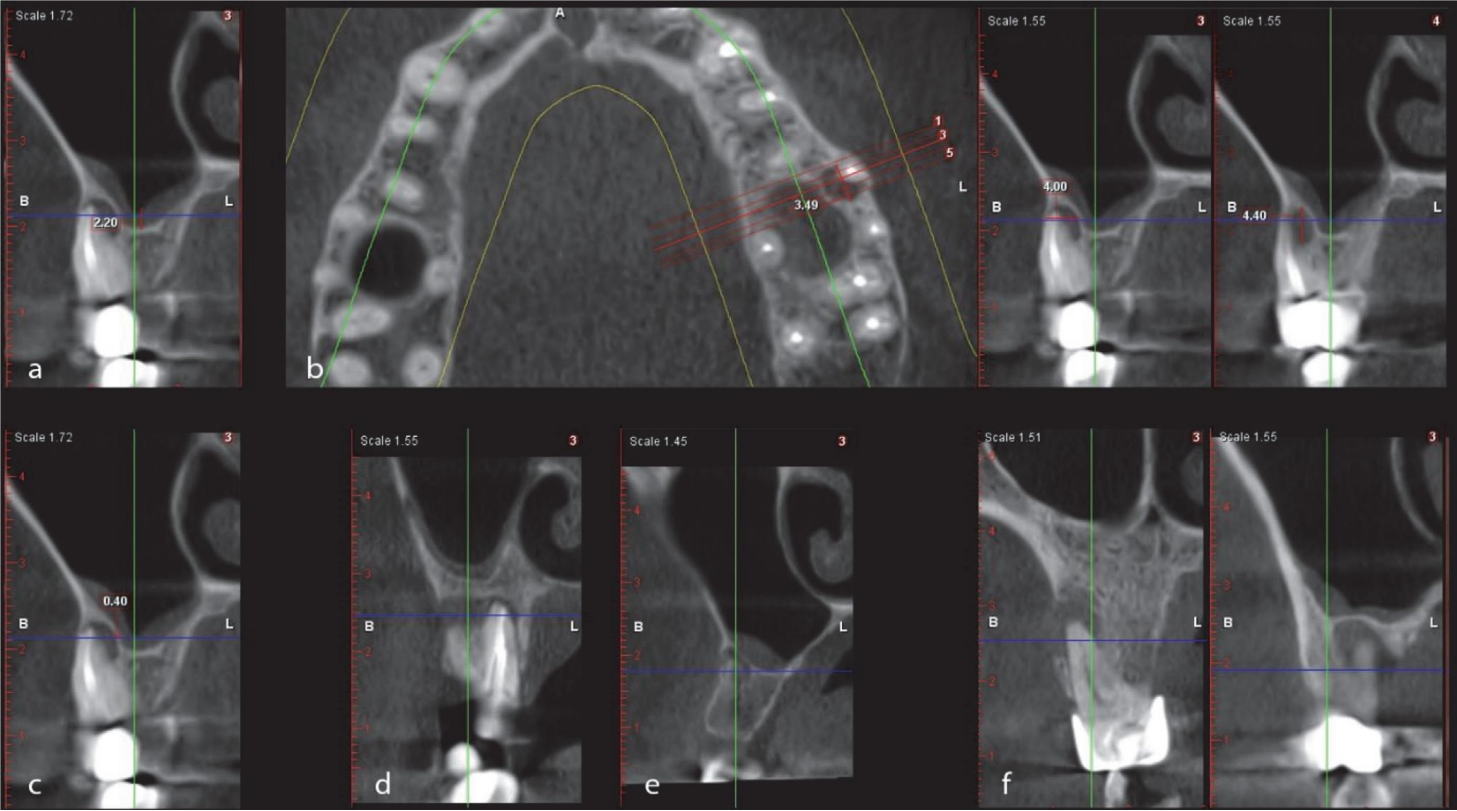
Representative CBCT images showing the radiographic variables evaluated in this study: (a) Schneiderian membrane thickness (SMT), (b) periapical lesion dimensions used for lesion volume estimation, (c) minimum bone thickness between the lesion and the sinus floor, (d) flat mucosal thickening, (e) round/curved mucosal morphology, and (f) alveolar bone plate perforation.

Image evaluation was performed primarily in coronal and sagittal views. The following variables were recorded: patient age; dimensions and volume of the periapical lesion; SMT; thickness of bone between the lesion and the sinus floor; mucosal morphology; presence of alveolar bone perforation; and the relationship of the lesion to the sinus floor, including the number of roots involved (if applicable). SMT was measured as the length of a line perpendicular to the sinus floor adjacent to the periapical lesion and recorded in millimeters (Figure 1a).

Periapical lesion dimensions were measured in three perpendicular directions (apicocoronal, mesiodistal, and buccolingual), and lesion volume (LV) was calculated as the product of these three dimensions and reported in mm^3^ (Figure 1b). Bone thickness (BT) was measured as the minimum bony thickness/distance between the periapical lesion and the sinus floor (Figure 1c). Mucosal morphology was categorized as flat or round/curved based on its appearance on CBCT (Figure 1d,e). Alveolar bone perforation (buccal or palatal) was assessed in cases with lesion extension and recorded as present/absent based on observation in axial and coronal views (Figure 1f).

### 2.8. Assessors and Reliability

CBCT assessment was performed by an oral and maxillofacial radiologist and a final‐year dental student. Before the formal image evaluation, the student underwent structured training and calibration under the supervision of the radiologist and completed multiple practice sessions to standardize the assessment approach. In addition, a second oral and maxillofacial radiologist was involved in the evaluation workflow. In cases of disagreement, the findings were reviewed jointly, and a consensus decision was reached through discussion with the specialist radiologists. Interobserver reliability for continuous measurements (SMT, BT, and LV) was assessed using the intraclass correlation coefficient (ICC). The ICC values were 0.77 for SMT, 0.83 for BT, and 0.72 for LV, indicating acceptable to good agreement overall.

### 2.9. Data Analysis

**Figure 2:**
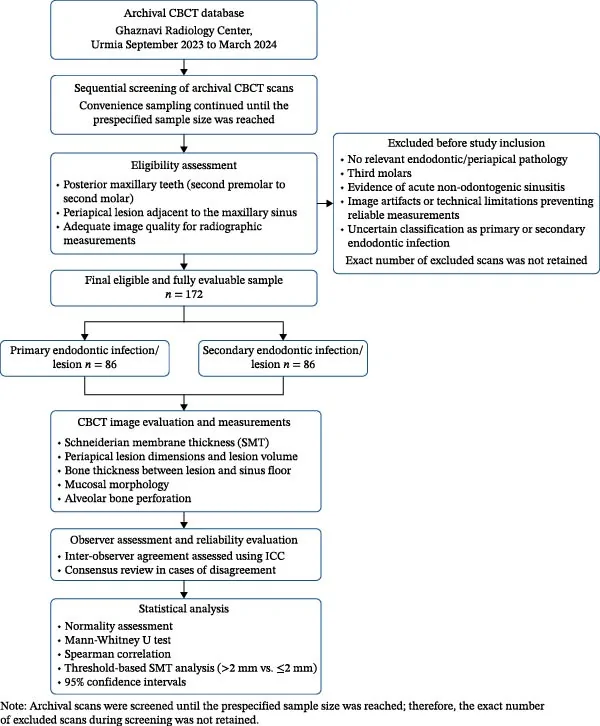
Workflow of case selection, eligibility assessment, CBCT evaluation, radiographic measurements, and statistical analysis.

Data were analyzed using IBM SPSS Statistics (version 22). Normality of quantitative variables was assessed using the Kolmogorov–Smirnov test. Between‐group comparisons were performed using the Mann–Whitney *U* test, and associations between quantitative variables were evaluated using Spearman’s correlation coefficient. SMT was additionally dichotomized as >2 mm versus ≤2 mm based on the commonly used radiographic threshold for Schneiderian membrane thickening. Statistical significance was set at a two‐sided *p* < 0.05. Ninety‐five percent confidence intervals (95% CIs) for continuous estimates and Spearman correlation coefficients were obtained using nonparametric bootstrap resampling with 10,000 resamples, whereas Wilson 95% CIs were calculated for proportions. No missing data were present among the 172 cases included in the final analyses; therefore, no imputation was required. The overall study workflow is summarized in Figure 2.

This study adheres to the STROBE guidelines for cross‐sectional studies.

## 3. Results

### 3.1. Study Sample and Baseline Characteristics

In this study, a total of 172 CBCT scans were evaluated and divided into two equal groups: the primary endodontic lesion/infection group (*n* = 86) and the secondary endodontic lesion/infection group (*n* = 86). The mean age was 43.53 ± 11.32 years in the primary group and 42.98 ± 11.02 years in the secondary group, with no significant difference between the groups (*p* = 0.697) (Table 1). Complete data were available for all variables in the 172 cases included in the final analysis.

**Table 1:** Baseline and radiographic characteristics in primary and secondary groups and overall sample.

Variable	Overall (*n* = 172)	Primary (*n* = 86)	Secondary (*n* = 86)	*p*‐Value
Age (years), mean ± SD	43.26 ± 11.14 [41.60–44.91]	43.53 ± 11.32 [41.19–45.97]	42.98 ± 11.02 [40.72–45.30]	0.697
SMT (mm), mean ± SD	3.72 ± 2.24 [3.40–4.06]	3.74 ± 2.01 [3.33–4.18]	3.69 ± 2.46 [3.22–4.25]	0.401
Bone thickness between lesion and sinus floor (mm), mean ± SD	1.38 ± 1.85 [1.11–1.66]	1.52 ± 1.98 [1.12–1.96]	1.23 ± 1.72 [0.88–1.62]	0.389
Periapical lesion volume (mm^3^), mean ± SD	165.99 ± 177.29 [140.71–193.59]	187.86 ± 201.37 [147.67–231.61]	144.12 ± 147.35 [115.10–177.30]	0.202
Mucosal morphology—flat, *n* (%)	111 (64.5%)	55 (64.0%)	56 (65.1%)	—
Mucosal morphology—round/curved, *n* (%)	61 (35.5%)	31 (36.0%)	30 (34.9%)	—
Alveolar bone plate perforation (any), *n*	62 (36.0%)	39 (45.3%)	23 (26.7%)	—
Buccal perforation, *n*	56 (32.6%)	36 (41.9%)	20 (23.3%)	—
Palatal perforation, *n*	6 (3.5%)	3 (3.5%)	3 (3.5%)	—

*Note:* Values in brackets represent 95% bootstrap confidence intervals. Test: Mann–Whitney *U*.

Abbreviation: SMT, Schneiderian membrane thickness.

### 3.2. Descriptive Radiographic Findings

In the overall sample, the mean SMT was 3.72±2.24 mm. The mean BT between the lesion and the sinus floor was 1.38± 1.85 mm, and the mean periapical LV was 165.99 ± 177.29 mm^3^. Regarding mucosal morphology, 111 cases (64.5%) showed a flat pattern, and 61 cases (35.5%) showed a round/curved pattern. Alveolar bone plate perforation was observed in 62 cases, including 56 buccal perforations and six palatal perforations (Table 1).

### 3.3. Normality of SMT Distribution

According to the Kolmogorov–Smirnov test, SMT was not normally distributed (*p* < 0.001); therefore, nonparametric tests were used in the analyses.

### 3.4. Comparison of SMT Between Primary and Secondary Groups

The mean SMT was 3.74± 2.01 mm in the primary group and 3.69 ± 2.46 mm in the secondary group. The mean difference between the groups was 0.06 mm (95% CI: −0.63 to 0.71 mm), and the difference was not statistically significant (Mann–Whitney *U* test: *Z* = −0.841, *p* = 0.401).

### 3.5. SMT by Type of Perforation

Among cases with perforation, the mean SMT was 3.59 ± 1.87 mm for buccal perforations (*n* = 56) and 4.62 ± 1.81 mm for palatal perforations (*n* = 6), with no significant difference observed (*Z* = −1.455, *p* = 0.146).

### 3.6. Correlation Analysis

No significant correlations were observed between SMT and age (*ρ* = 0.089, 95% CI: −0.060 to 0.238, *p* = 0.248), LV (*ρ* = 0.101, 95% CI: −0.051 to 0.249, *p* = 0.189), or BT (*ρ* = −0.089, 95% CI: −0.234 to 0.054, *p* = 0.245) (Table 2).

**Table 2:** Spearman correlations between SMT and age, lesion volume, and bone thickness.

Variable		Age	Lesion volume	Bone thickness
SMT	Spearman correlation coefficient	0.089	0.101	−0.089
	95% CI	−0.060 to 0.238	−0.051 to 0.249	−0.234 to 0.054
	‐*p*‐Value	0.248	0.189	0.245

*Note:* 95% CIs were estimated using nonparametric bootstrap resampling (10,000 resamples). Test: Spearman correlation coefficient.

Abbreviation: SMT, Schneiderian membrane thickness.

In the threshold‐based analysis (Table 3), SMT > 2 mm was observed in 76 of 86 cases (88.4%) in the primary group and 74 of 86 cases (86.0%) in the secondary group, with no significant difference between the groups (chi‐square, *p* = 0.819).

**Table 3:** Threshold‐based comparison of SMT between primary and secondary endodontic lesions.

SMT category	Primary (*n* = 86) *N* (%) [95% CI]	Secondary (*n* = 86) *N* (%) [95% CI]	Total (*n* = 172) *N* (%) [95% CI]	*p*‐Value
>2 mm	76 (88.4%) [79.9–93.6]	74 (86.0%) [77.2–91.8]	150 (87.2%) [81.4–91.4]	0.819
≤2 mm	10 (11.6%) [6.4–20.1]	12 (14.0%) [8.2–22.8]	22 (12.8%) [8.6–18.6]	—

*Note:* 95% CIs for proportions were calculated using the Wilson method. Test: Chi‐square test.

Abbreviation: SMT, Schneiderian membrane thickness.

## 4. Discussion

Maxillary sinusitis is one of the most common head and neck infections and, although it may be chronic and asymptomatic, can have significant clinical consequences. Evidence suggests that the proximity of periapical lesions to the maxillary sinus can be associated with changes in the sinus mucosa [7].

CBCT was used in the present study because it allows for three‐dimensional assessment of the anatomical relationships between the maxillary posterior teeth, periapical lesions, and the maxillary sinus and reduces the limitations of two‐dimensional methods (including overlapping structures) [32]. Based on a systematic review, the mean SMT is ~2 mm in CBCT studies of patients without symptomatic sinusitis [33].

The main finding of the present study was that SMT did not differ significantly between primary and secondary endodontic lesion/infection groups (*p* = 0.401). This is of clinical importance, as many studies have suggested an association between endodontic pathology and increased SMT, but the relative role of primary versus secondary infection remains controversial [6, 7, 17, 18, 31, 34]. Some studies have reported a greater association of Schneiderian membrane thickening with primary infection [7, 18], while others have suggested a stronger role for secondary/posttreatment infection [17]. Some reports have also reported a positive association between endodontic lesions and chronic rhinosinusitis [35]. However, the results of studies are not consistent, and some studies have not reported a significant association between the presence of endodontic lesions and increased membrane thickness [36]. Therefore, the lack of a significant difference between the primary and secondary groups in the present study could reflect differences in the sample, selection criteria, lesion severity, proximity to the sinus floor, or differences in measurement protocols.

In secondary analyses, no significant association was observed between SMT and age (*p* = 0.248), LV (*p* = 0.189), and BT between the lesion and the sinus floor (*p* = 0.245). The lack of an age‐related association with SMT is consistent with some studies [37], but there are also studies that have reported increased thickness at older ages (especially >60 years) [7, 31, 38]. The lack of association of SMT with LV is also consistent with some reports [39], whereas in some studies, LV—especially in secondary lesions—has been associated with increased SMT [31, 40]. Regarding BT, our findings are consistent with some reports [31], but some studies have suggested a role for cortical plate changes/bone resorption in the association with increased SMT [9].

The threshold‐based analysis (>2 mm) yielded results consistent with the main continuous–variable analysis, further supporting the absence of a significant difference in SMT between primary and secondary endodontic lesions.

In terms of mucosal morphology, a flat pattern was more common in the present sample, and this finding is comparable to some CBCT‐based reports [40]. Morphological segmentation of the Schneiderian membrane can be helpful in interpreting the etiology of sinus involvement [41]. Regarding perforation, buccal perforation was more common than palatal, and there was no significant difference in SMT between buccal and palatal perforations (*p* = 0.146). This comparison should be interpreted with caution because of the small number of palatal perforations.

From a clinical perspective, the present findings suggest that SMT on CBCT should not be interpreted in isolation as a marker for distinguishing primary from secondary endodontic infection. Although Schneiderian membrane thickening adjacent to posterior maxillary teeth may support the possibility of odontogenic sinus involvement, the absence of a significant difference between the two groups in the present study indicates that the type of endodontic infection alone may have limited discriminatory value in this context. Therefore, CBCT findings should be interpreted together with treatment history, lesion extent, the number of roots involved, the relationship of the lesion to the sinus floor, and the patient’s clinical and sinonasal findings. In practical terms, the role of CBCT may lie less in inferring whether an adjacent lesion is primary or secondary based on membrane thickness alone and more in identifying possible odontogenic sinus involvement, estimating disease extent, and supporting treatment planning or referral when sinus‐related findings are clinically relevant.

## 5. Limitations

This study has several limitations. First, because of its retrospective cross–sectional design and the use of archival CBCT scans, causal inference cannot be established. Second, potentially relevant confounding variables, such as periodontal status, smoking habits, systemic conditions, allergy history, and previous sinus pathology, were not comprehensively available for assessment or adjustment and may have influenced SMT. Third, classification of lesions as primary or secondary relied on radiographic findings and treatment history rather than microbiological confirmation; therefore, some degree of misclassification bias cannot be excluded. These limitations should be considered when interpreting the present findings, and future prospective studies with broader clinical data and microbiological confirmation are recommended.

## 6. Conclusions

In this retrospective cross–sectional CBCT study, SMT was not significantly associated with the type of endodontic lesion/infection (primary vs. secondary). No significant associations were also found between SMT and age, LV, or BT between the lesion and the sinus floor. Because of the observational cross–sectional design, these findings do not support causal inference.

## Funding

The authors received no specific funding for this work.

## Conflicts of Interest

The authors declare no conflicts of interest.

## Data Availability

The data that support the findings of this study are available from the corresponding author upon reasonable request. However, for privacy reasons, no individual data allowing identification of participants can be provided.
